
JOURNAL INFORMATION
==============================
NLM Title Abbreviation: Buffalo Med J
NLM Journal ID: 0027534

Buffalo Medical Journal

ISSN: 1040-3817
Publisher: Biomedical Journal Digitization Project

ARTICLE INFORMATION
==============================
PMCID: PMC8726856
PMID: 36885261
Article version: 1
Subjects: Miscellany

Miscellany

Print publication date: 1908 Oct
Volume: 64
Issue: 3
First page: 178
Last page: 178
License: This work is free of known copyright restrictions. For more information, please see PMC Back Issue Digitization.
License URL: https://creativecommons.org/publicdomain/mark/1.0/

==============================

General Surgery. By Ehrich Lexer, M.D., Professor of Surgery in the University of Koenigsberg. American edition edited by Arthur Dean Bevan, M.D., Professor and head of the Department of Surgery, Rush Medical College, (University of Chicago). An authorised translation of the second German edition by Dean Lewis, M.D., Assistant Professor of Surgery, Rush Medical College. Octavo, pp. 1041. With 449 illustrations and two colored plates. New York and London: D. Appleton & Co.

---

Fourth Annual Report of the Henry Phipps Institute for the study, treatment and prevention of tuberculosis. February 1, 1906 to February 1, 1907. Edited by Joseph Walsh, A.M., M.D.

---

Transactions of the thirty-fourth annual meeting of the Oregon State Medical Association held at Portland, Oregon, July 1, to 3, 1908. William House, M.D., secretary.

---

The Cure of Rupture by Paraffin Injections by Charles C. Miller, M.D. Small 12 mo, pp. 81. Published by the Author, 70 State St., Chicago. (Prepaid $1.00.)

---

### MISCELLANY.

---

THE State Civil Service Commission will hold examinations on October 17, 1908, for the following positions: Assistant in Clinical Psychiatry, Pathological Institute, $2,000; Health officer for Wells, Hamilton County; physician, sixth grade for hospitals and institution, $900, and maintenance; Veterinarian State Department of Agriculture and Health, $5.67 a day when employed.

The last day for filing applications for these positions is October 10. Full information and application forms for any of these examinations can be obtained by postal card request to the Chief Examiner of the Commission at Albany. Charles S. Fowler, Chief Examiner.

---

THE United States Civil Service Commission announces an examination on October 21, 1908, at the usual places, to secure eligibles from which to make certification to fill a vacancy in the position of aid (male), in the Division of Physical Anthropology, U. S. National Museum, at $50.00 a month, and vacancies requiring similar qualifications as they may occur.

---

**FOR SALE**

My residence, 189 Fourteenth Street, has been occupied by a physician for twenty years, and is well adapted for such purposes. Reasonable terms.

Address, DR. E. T. SMITH, 189 Fourteenth Street, Buffalo, N. Y.
